# Risk Profiles of Korean Adolescents in Relations With Contextual Factors: Implications for Multi-Tiered Systems of Support

**DOI:** 10.3389/fpsyt.2022.796928

**Published:** 2022-03-16

**Authors:** Dongil Kim, Jin Hyung Lim

**Affiliations:** Department of Education, Seoul National University, Seoul, South Korea

**Keywords:** youth at-risk, risk profiles, contextual factors, MTSS, KCYPS 2018

## Abstract

**Introduction:**

Although prior studies have supported the effectiveness of Multi-Tiered Systems of Support (MTSS) on addressing social, emotional, behavioral, and academic challenges faced by youth at-risk, educators using MTSS often do not consider contextual factors which may also influence youth at-risk and the interventions targeting them. This study thus aimed to identify youth at-risk who should be referred to targeted instructions within MTSS by examining the risk profiles of Korean adolescents. Based on the identified risk profiles, we also tried to investigate the effect of contextual factors on deciding youth at-risk and confirm whether and/or what contextual factors should be considered when implementing targeted interventions for them.

**Method:**

To accomplish the research goal, a latent profile analysis on risk factors of Korean adolescents was performed, using the first year data of “Korean Children and Youth Panel Study (KCYPS) 2018.”

**Results:**

Four risk profiles were identified, using low academic motivation, low academic behavior, attention deficit, aggression, social withdrawal, and depression as indicators: the high risk, M-SEB (Moderate-social, emotional, & behavioral) risk, M-ACA (Moderate-academic) risk, and low risk group. The covariates of this study, home and school environmental variables, worked as predictors of adolescents included in the high group.

**Conclusion:**

The results of this study suggest students in the high risk group (16.8%) should be given targeted instructions combining academic and SEB support within MTSS so as to prevent negative outcomes in the future among all adolescents. Those instructions need to be planned with consideration of contextual factors accompanied by teacher's careful understanding of social dynamics surrounding each student.

## Introduction

Adolescence involves dramatic social, psychological, and physical changes, which have great influences on social and career adjustment in adulthoods (1). This phase is also important since a great number of adolescents can face diverse risks that may prevent normal development and lead to academic failure, mental health problems, and maladjustment in society (2). Thus, it is highly necessary to monitor developmental trajectories of youth and identify whether they have certain risk factors that may result in significant problems in the future. In other words, early identification and intervention to support youth at-risk should be one of the primary goals of secondary education.

### Youth At-Risk

The concept “youth at-risk” has been defined in several ways. According to Resnick and Burt (3), youth at-risk is defined as adolescents with negative antecedent conditions creating vulnerabilities, combined with the presence of specific negative behaviors or experiences that are likely to lead to more serious long-term health consequences. Similarly, Evans (4) stated youth at-risk as adolescents who are unlikely to achieve independent adulthood due to maladjustment to school life, estimating about 16% of all adolescents as youth at-risk. Dryfoos (5) also identified adolescents (age between 10 and 17) at risk who have risk markers such as delinquency, substance abuse, or academic suspension, and 25% of all youths are designated to be at high risk. Although the definition and specific proportion deciding youth at risk were not exactly identical across researchers, it was agreed that youth at-risk experiences risks that may lead to other negative outcomes in the long run across social, emotional, behavioral, and academic domains.

For an academic domain, youth at-risk tend to experience significant distress and marginalization in classrooms because of push for testing outcomes and academic accountability (6). They are more likely to fail in academic achievement assessments and less likely to meet standards of general curriculum than their peers (6). In reciprocal relationships with academic skills, academic motivation is also one of the most typical characteristics of youth at-risk. Academic motivation is able to be generated by students having a goal of gaining a rich understanding of experiences through learning (7). However, it was frequently reported that students at-risk with the accumulated academic helplessness do not understand the value of studying (8). It is also important to note low academic motivation is strongly associated with low academic behavior which is defined as behaviors that promote one's ability to be prepared for, participate in, and benefit from an academic instruction (9). Since students with low academic motivation and behavior can develop serious problems such as academic failure and dropping out of school (7), supporting those students with effective intervention programs is highly recommended.

Youths at-risk also have been reported to experience attention deficits. Students having difficulties focusing on a certain task for an extended period of time predicts not only maladjustment to school-life but to drug use and addictive behavior in the future (10). If without appropriate educational intervention, these symptoms often result in difficulties from work and interpersonal relations, low self-esteem, anxiety, and emotional liability in adulthood (11). As students with attention deficit often have comorbidity with hyperactivity, aggression also presents similar patterns in developmental trajectories of youth. According to Sharma and Marimuthu (12), aggression in the age of 10–16 was highly related to hyperactivity, low academic performance, peer delinquency, and drug abuse. Therefore, aggression along with attention deficit can be important indicators for identifying adolescents at-risk in academic and behavioral domains.

Some youths-at-risk are characterized as socially withdrawn, spending most of their time alone and on the periphery of the social settings due to shyness or social anxiety (13). Social withdrawal has been shown to be stable from ages 5 to 11 years and so on (14), which can be a risk factor for psychosocial maladjustment since it is deeply interrelated with negative self-esteem, anxiety, depression, and peer rejection (15, 16). In addition, depression can also be the risk factor of the emotional domain, as adolescent depression has been highly correlated with adverse psychosocial and academic outcomes and increased incidence of substance abuse and suicide (17). According to Field et al. (18), depression in adolescence is deeply associated with relationships with parents, peers, lifestyle, and emotional wellbeing. Thus, depression is also qualified to be included in risk factors predicting adverse outcomes in the future as well as being affected by surrounding environments.

Throughout previous studies, the abovementioned externalizing (e.g., attention deficit, aggression) and internalizing (e.g., social anxiety, depression) risks are also highly correlated with effortful control, which refers to the ability to regulate cognition, emotion, and behavior (19). As this neurocognitive variable has been identified as a contributor to future outcomes across diverse domains, along with externalizing and internalizing challenges (19), a lack of this competency during adolescence would be able to predict adverse educational attainment of adulthood (20). Hence, neurocognitive difficulties may also deteriorate negative outcomes of youth at risk.

Previous studies have also supported that environmental factors significantly affect student's diverse risks across social, emotional, behavioral, and academic domains. According to Lim (21), home environments, including interaction with parents and school environments, including relationships with peers and teachers, had statistically significant influences on adolescent's level of mental health regardless of whether they experience low academic achievement. Specifically, students experiencing low-quality relationships with their parents, peers, and teachers tend to report higher risks in internalizing problems such as anxiety, depression, and suicidal impulse. Since adolescent's mental health problems predict school adjustment in the long term (22), it is reasonable to conclude that home and school environmental factors surrounding students are critical determinants of their school adjustment. Furthermore, Kim and Lim (23) also suggested that identical contextual factors are likely to affect adolescent's self-concept in various domains. Considering that the self-concept reflects one's own belief of oneself in social, familial, and academic contexts, which strongly affect life satisfaction and overall wellbeing, home and school environmental variables should be carefully examined and regulated in order for students to maintain healthier lives. Therefore, it is reasonable to assume that those ^*^ environmental factors are the vital contributors to the diverse challenges faced by adolescents.

### MTSS to Support Youth At-Risk

Youth at-risk students need to be supported across academic, social, emotional, and behavioral domains in order not to experience adverse consequences in their adulthood adjustment. There were numerous attempts to support youth at-risk within school settings, and among them, the Multi-Tiered System of Support (MTSS) has been the representative model of early identification and systematic intervention targeting students at-risk. MTSS is a comprehensive framework designed to address the interplay of social, emotional, behavioral, and academic functioning and adaptation in the classroom (24, 25), which encompasses every kind of challenge students face. It emphasizes students' responsiveness to intervention and requires both universal and incrementally intensive strategies that encompass the students with diverse severity of difficulties (26) by providing more intensive strategies to students who do not respond to general instructions (24). To be specific, it is usually configured in pyramid-shaped three intervention levels: Tier 1 (universal instruction) is for the universal support providing strategies that are applied to all students as a foundation for specialized interventions; Tier 2 (selective instruction) consists of selective interventions to focus on individuals who can be classified as “students at-risk” and whose needs are not adequately met by Tier 1 approaches, which typically includes about 10–20% of all students; and Tier 3 (individualized instruction) indicates targeted strategies individualized to the needs of each student and generally for the 5–7% of students who do not respond to former interventions (27). Adopting a preventive approach that involves the early identification and provision of services before their problems are manifested and are identified as a disability in a student's functioning (28), MTSS is now widely accepted to initiate school-wide prevention and intervention model for students facing various risks.

Having lots of benefits, a significant limitation of MTSS widely agreed on is that its focus is primarily on intervention intensity and not tentative variables which contribute to student's add adoption (26, 29). In a traditional MTSS framework, the provision of educational services is solely determined by student's progress in targeted performances. It suggests the movement to the next level of intervention with a more intensive strategy if a student is not responsive to a less intensive level of instructions (30). However, as we have reviewed that risk factors of adolescents are significantly impacted by home and classroom environments surrounding each student (21–23), the response to intervention is also highly likely to be affected by identical contextual variables (26, 29). For example, according to Farmer et al. (27), teachers are familiar to conclude that the instruction was ineffective or the student is resistant to the instruction based on the lack of student progress after the instruction. In contrast, they often do not assume other contextual variables operated to prevent student's progress despite the high level of effectiveness of interventions, which leads them to subsume the educational services are sufficient even when instructional strategies are not fully adapted to the needs of each student (27). This does not indicate that MTSS is flawed or ineffective. Instead, it shows the necessity of educators considering contextual and ecological factors when planning instructions from the multi-tiered system since students' competencies in social, emotional, behavioral, and academic domains tend to develop as a whole in relation to those factors (31).

### The Current Study

Although the abovementioned drawback of MTSS seems convincing, there was a lack of efforts to empirically demonstrate exactly what ecological factors significantly decide the challenges of youth at-risk. In this sense, the current study as supplementation of traditional MTSS was planned to confirm whether or what contextual factors can have significant impacts on deciding youth at risk. To accomplish this research goal, we conducted a latent profile analysis (LPA) to determine who are able to be identified as youth at-risk and should be referred to more intensive instructions within MTSS. The indicators for the LPA encompass social, emotional, behavioral, and academic challenges due to the aim of MTSS dealing with all kinds of problems faced by students (24). After deciding youth at-risk through LPA, we verified the effect of contextual variables on the decision of youth at-risk, compared to other latent profiles. As Kim and Lim (23) stated that the primary contexts surrounding adolescents could be signified as home and school environments, and Farmer et al. (32) suggested that dynamic relationships with significant others surrounding each student affect the effectiveness of interventions, home environmental variables (e.g., parenting attitudes) and school environmental variables (e.g., relationships with peers and teachers) were included as contextual factors.

In reviewing related research, Cho et al. (33) attempted to address the latent profiles based on diverse risk factors of Korean adolescents. However, the data originated from the teachers' perception of the characteristics of youth at-risk, not from the self-report of challenges. Furthermore, there were no studies identifying the relationships of risk profiles with contextual factors. Therefore, the present study is highly valuable as we successfully identified youth at-risk based on nationwide data [in this case, data from Korean Children and Youth Panel Survey (KCYPS) 2018] reported by students themselves and verified environmental variables that predict those youths. Through the findings of this research, we anticipate that the overall effectiveness of tiered instructions within MTSS can be enhanced by informing clinicians and educators of the most appropriate services as well as that the proportion of students inadvertently placed in a more intensive tier without receiving adaptive interventions in a lower tier may decrease.

To sum up, using the KCYPS data, this study was designed to identify the risk profiles of Korean adolescents across diverse domains and the impact of contextual factors on those profiles. In addressing the study purpose, the following research questions are raised:

RQ1: Who can be identified as youth at-risk who should be referred to selective instructions within MTSS?

RQ2: Whether and/or what contextual factors have significant impacts on deciding youth at-risk?

## Methods

### Sample

Data for the current study was collected from the “Korean Children and Youth Panel Survey (KCYPS) 2018” which is longitudinal data conducted by National Youth Policy Institute in South Korea. Starting in 2018, KCYPS 2018 is designed to keep track of the educational background and characteristics of students in elementary and middle schools. We used the first and second year data of KCYPS 2018 with information of 2,590 middles school first graders in 2018. In regard to the demographic composition of the sample, the percentage of male participants were 54.2, while that of female participants were 45.8. 45.1% of the students attended schools in urban regions, 40.7% were in suburban districts, and 14.2% were in rural regions.

### Variables

#### Indicators: Risk Factors

To assess levels of risks that participants counter and identify the risk profiles of Korean adolescents, we selected six variables among the first-year data of KCYPS 2018 as indicators; academic motivation, academic behavior, attention deficit, aggression, social withdrawal, and depression. For the academic motivation scale, the higher score indicates that the participant has a lower level of academic motivation and it includes four items such as “I do not know why I should study hard.” and “I do not enjoy studying.” The higher score of academic behavior scale shows that the responder is less likely to be engaged in academic-related behaviors, such as classroom activity or plan for their own learning, with four items. Both academic motivation and behavior scales were validated in Bak et al. (34) by sampling 593 elementary and secondary school students in Korea. The higher score of attention deficit scale means the participant has more difficulties concentrating on one task for an extended period of time. A total of seven items of this scale include “I do not want to finish my homework that needs concentration for a long time.” and “I feel discomfort when I have to sit quietly while studying.” For the aggression scale, students with higher score indicates they are more likely to be in high-temper. The six items for the aggression level include “I often disturb what someone else is doing.” and “I often fight with other friends for minor reasons.” Both attention deficit and aggression scales were validated through Cho and Lim (35) collecting data from 457 4 to 6^th^ graders in Korea. The social withdrawal scale was developed and standardized by Kim and Kim (36) based on the data from 518 individuals from 5 to 8^th^ grades. The higher score of the social withdrawal scale means the participants are more reluctant to show themselves or present their feelings in front of other people. For example, statements such as “I often feel shy.” and “I do not want to express myself in front of many people.” are included in the scale with a total of five items. Lastly, the higher level of depression indicates that students are more lethargic and feel more depressed. Ten items of depression include “I do not have interests in every circumstance.” and “I want to die,” which were designed and validated by Kim et al. (37). Every scale selected in order to measure abovementioned risk factors was designed to be 4-Likert scales (1 = strongly disagree, 4 = strongly agree), and the reliability of each scale was also satisfactory (Table 1).

**Table 1:** The reliability of scales used to measure study variables.

	**Variables**	**Number of items**	**Cronbach's a**
Risk factors	Low academic motivation	4	0.905
	Low academic behavior	4	0.785
	Attention deficit	7	0.820
	Aggression	6	0.839
	Social withdrawal	5	0.874
	Depression	10	0.922
Home environment	Parent warmth	4	0.913
	Parent acceptance	4	0.789
	Parent consistency	4	0.804
School environment	Peer relationship	13	0.852
	Teacher relationship	14	0.912

#### Covariates: Contextual Factors

Contextual factors that presumably influence diverse difficulties faced by adolescents were incorporated in our study as covariates in order to determine whether they predict risk profiles each individual would show. These predictors were home and school environmental variables from the first-year data. For home environmental variables, three scales related to parenting attitudes were included; parental warmth, acceptance, and consistency. In the parent warmth scale, the higher score of warmth indicates that parents are more likely to keep close relationships with their children by expressing their love and kindness. Four items were included in this scale with “My parents always express love for me” for an example. The parental acceptance scale consists of four items with student's self-report of conceptualizations that their parents feel satisfactory with their children. Since the questions are in negative statements, such as “My parents make me think I am unnecessary.” and “My parents are never satisfied with what I am doing,” we inversely coded the response of each student to make the higher score indicate a higher level of acceptance. The parental consistency scale shows the degree of directions of parents to their children being consistent in diverse contexts. As four items of this scale also are in negative statements (e.g., “My parents often change rules for me.”), the answer of these items were inversely coded. All of these scales were developed in Kim and Lee (38) and predictive validity was also confirmed based on the data of 507 middle school students in Korea.

School environmental variables consist of two independent scales; peer relationship and teacher relationship. A peer relationship scale shows how a student makes relationships with classmates, with eight items for positive relationships (e.g., “I can tell my secrets to my friends.”) and five items for negative relationships (e.g., “My friends do not care for my difficulties.”). To make the higher score of this scale indicate more agreeable peer relationships, we inversely coded the answers of items negatively stated. This scale was validated in Bae et al. (39) by sampling 393 middle and high school students in Korea. Lastly, a teacher relationship scale consists of 14 items that shows whether teachers are credible, available, acceptable, and sensitive to the needs of each student (e.g., “My teacher respects my opinion,” “My teacher waits for me until I answer the question.”). The higher the score of this scale, the more the students are likely to have good relationships with their teachers. Kim and Kim (40) validated this scale based on the data of 2,056 individuals from elementary and middle school in Korea. All home and school environmental variables were constructed to be 4-Likert scales (1 = strongly disagree, 4 = strongly agree), and the reliability of each scale was also satisfactory (see Table 1).

### Statistical Analysis

For the statistical analysis, we adopted Latent Profile Analysis (LPA) as a primary research method. LPA enables researchers to capture substantial groups of people whose responses to certain indicators are similar and to identify unobserved homogeneity or heterogeneity in a population (41). LPA is often called a method with a person-centered approach due to its focus on relationships between people, instead of relationships between variables (42).

In advance of performing LPA, processed in the statistical program SPSS 22.0, descriptive statistics and correlation analyses were conducted (see Tables 2, 3). These analyses were to confirm the general tendencies of raw data and whether the normality assumption for LPA is fulfilled. The normality assumption is fulfilled if the absolute value of skewness is lower than 2 and that of kurtosis is under 7 (21). According to Table 2, the descriptive statistics of all variables inserted in LPA successfully fulfilled the normality assumption. Furthermore, Table 3 shows that all coefficients of the correlation analysis were statistically meaningful (*p* < 0.001 for each correlation, *p* < 0.05 for family-wise error rate), and that all risk factors had negative relationships with contextual factors.

**Table 2:** The descriptive statistics of study variables.

	**Variables**	**Mean**	**Standard deviation**	**Skewness**	**Kurtosis**
Risk factors	Low academic motivation	1.96	0.74	0.53	−0.11
	Low academic behavior	1.95	0.64	0.30	−0.29
	Attention deficit	2.17	0.56	−0.06	−0.02
	Aggression	1.91	0.59	0.11	−0.54
	Social withdrawal	2.15	0.75	0.15	−0.61
	Depression	1.80	0.64	0.62	0.03
Home environment	Parental warmness	3.37	0.58	−0.54	−0.10
	Parental acceptance	3.23	0.62	−0.79	0.79
	Parental consistency	3.00	0.64	−0.26	−0.07
School environment	Peer relationship	3.13	0.43	−0.09	0.29
	Teacher relationship	2.81	0.50	−0.15	1.03

**Table 3:** A correlation analysis of study variables.

	**1**	**2**	**3**	**4**	**5**	**6**	**7**	**8**	**9**	**10**
1	1									
2	0.643	1								
3	0.464	0.504	1							
4	0.399	0.399	0.605	1						
5	0.277	0.299	0.304	0.396	1					
6	0.409	0.378	0.391	0.596	0.552	1				
7	−0.316	−0.302	−0.252	−0.324	−0.195	−0.368	1			
8	−0.299	−0.257	−0.278	−0.347	−0.152	−0.348	0.458	1		
9	−0.343	−0.295	−0.337	−0.412	−0.256	−0.380	0.417	0.482	1	
10	−0.307	−0.345	−0.277	−0.381	−0.338	−0.381	0.343	0.306	0.299	1
11	−0.338	−0.342	−0.307	−0.297	−0.236	−0.312	0.374	0.210	0.237	0.390

*1, low academic motivation; 2, low academic behavior; 3, attention deficit; 4, aggression; 5, social withdrawal; 6, depression; 7, parent warmness; 8, parent acceptance; 9, parent consistency; 10, peer relationship; 11, teacher relationship*.

For conducting LPA, we followed a three-step approach originated from Asparouhov and Muthen (43), using the statistical program Mplus ver. 8. The first step is to determine how many latent profiles fit the data best, only including the indicators to prevent the covariates variables from affecting the classification of the latent profiles. To decide the number of profiles, AIC (Akaike's Information Criterion), BIC (Bayesian Information Criterion), saBIC (sample-size adjusted Bayesian Information Criterion) were utilized, and the lower values of those indicators indicate the better fit. In addition, we used LMR (Lo-Mendell-Rubin) and BLRT (Bootstrapped Likelihood Ratio Test) statistics, which compare model fits by testing significance level of difference between the current profile classification (*N* = k) and one less profile (*N* = k-1). If *p*-values of LMR and BLRT are below 0.05, the current model fit (*N* = k) has improved from the former model (N=k-1). Lastly, we also used entropy which is a value that represents the clarity of each profile membership, ranging from 0 to 1. Entropy that is below 0.60 indicates about 20% of the participants were mistakenly classified in profiles, whereas that over 0.8 shows the profiles of over 90% of participants were successfully determined (44). Entropy about 0.7 is generally accepted in LPA studies. For the second step, the most likely class is created, where every individual with the highest membership probability is assigned to a profile. In the third step, the contextual factors are incorporated to the model so as to conduct a multinomial logistic regression analysis within the Mplus program.

## Results

### Risk Profiles of Adolescents

To solve the first research question, the latent profile model fit indicators were compared stepwise as Table 4 shows. The values of AIC, BIC, saBIC, and entropy decreased as the number of latent profiles was progressively added. However, the significance levels of LMR and BLRT were above 0.05 in a 5-profile model, which shows that the 4-profile model has the best fitness among all models. In addition, the entropy for the 4-profile model was 0.75, showing the acceptable level of clarity across the four latent profiles. As the 4-profile model also showed that every individual with the highest membership probability is assigned to a profile (see Table 5), we determined the number of latent profiles as four.

**Table 4:** A latent profile analysis to identify risk profiles of Korean adolescents.

	**AIC**	**BIC**	**saBIC**	**Entropy**	**LMR (*p*)**	**BLRT (*p*)**	**Percentage for each profile**				
							**1**	**2**	**3**	**4**	**5**
1-profile	30736.35	30806.67	30768.54	-	-	-	100.0				
2-profile	26998.05	27109.38	27049.01	0.81	0.000	0.000	58.8	41.2			
3-profile	26132.63	26284.97	26202.36	0.76	0.001	0.000	50.4	27.4	22.2		
4-profile	25704.73	25898.09	25793.24	0.75	0.003	0.000	41.9	28.6	16.8	12.8	
5-profile	25445.56	25679.93	25552.84	0.75	0.274	0.000	38.3	27.6	18.9	9.0	6.2

**Table 5:** Average latent profile probabilities for most likely latent profile membership (row) by latent profiles (column).

	**Profile 1**	**Profile 2**	**Profile 3**	**Profile 4**
Profile 1	0.919	0.000	0.040	0.041
Profile 2	0.000	0.863	0.019	0.118
Profile 3	0.070	0.015	0.788	0.127
Profile 4	0.026	0.060	0.065	0.848

**Figure 1:**
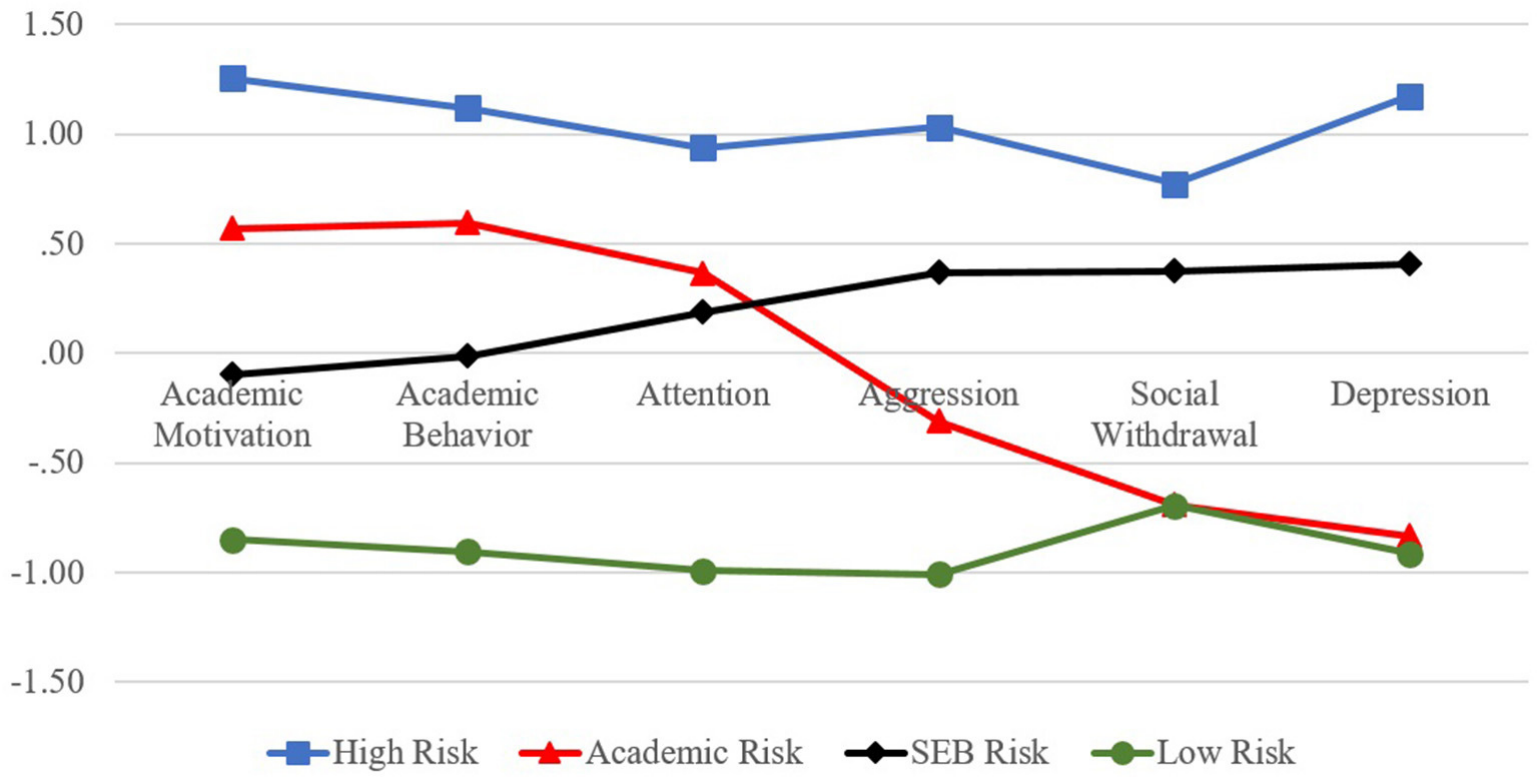
Risk profiles of Korean adolescents.

Figure 1 visualizes four risk profiles in a line graph. The horizontal axis represents the categories of diverse risks faced by adolescents, while the vertical axis indicates the mean values of standardized scores for each indicator. Each profile had approximately 16.8% (434 individuals; Group 1), 12.8% (331 individuals; Group 2), 41.9% (1,084 individuals; Group 3), and 28.6% (741 individuals; Group 4) of the total sample. Group 1 was named a “high risk” group since students in this profile showed the highest mean values across all indicators, whereas we called Group 4 a “low risk” group as they had the lowest mean values. Group 2 and 3 were named “moderate risk” groups because their mean values were located between high and low risk groups, but their patterns were slightly different from each other. In group 2, the mean values of aggression, social withdrawal, and depression that show social-emotional-behavioral (SEB) risks are higher than those of academic motivation and behavior which indicate academic risks. In group 3, however, the mean values of academic (ACA) risks are higher than those of social-emotional-behavioral (SEB) risks. Thus, we can regard group 2 as a “moderate-SEB (M-SEB) risk” group and group 3 as a “moderate-ACA (M-ACA) risk” group. Among four groups, students in the high risk group are able to be identified as youth at-risk since those students showed the highest levels of risks across all indicators, and the percentage of students included in this group was 16.8%, which was identical with the typical proportion of students (15–25%) who should be referred to selective interventions within MTSS (4, 45).

### Contextual Factors of Risk Profiles

A multinomial logistic regression was conducted in order to figure out whether and/or exactly what contextual factors have significant impacts on deciding youths at-risk who were included in the high risk group (see Table 6). When setting a low risk group as the reference, all home and school environmental variables had statistically significant impacts on falling into a high risk group. To be specific, the possibility to be included in a high risk group can be 43.1% lower when one level of parental warmth is increased, 55.4% lower when one level of parental acceptance is increased, 85.8% lower when one level of parental consistency is improved, 94.1% lower when one level of peer relationship is enhanced, and 85.9% lower when one level of teacher relationship is improved. If we set M-ACA risk group as a reference, the possibility to become a high risk group can be 47.2% lower when one level of parental warmth is improved, 43.0% lower when one level of parental acceptance is enhanced, 50.2% lower when one level of parental consistency is increased, 88.2% lower when one level of peer relationship is improved, and 64.3% lower when one level of teacher relationship is enhanced. Lastly, when setting a M-SEB risk group as a reference, the possibility to become a high risk group can be 23.8% lower when one level of parental acceptance is improved, 54.0% lower when one level of parental consistency is enhanced, 64.3% lower when one level of peer relationship is increased, and 67.4% lower when one level of teacher relationship is improved, while parental warmth and consistency did not have significant impacts.

**Table 6:** Tests of multinomial logistic regressions using the three-step procedure.

	**High risk VS. Low risk**		**High risk VS. M-ACA risk**		**High risk VS. M-SEB risk**	
	**Odds ratio**	***P-*value**	**Odds ratio**	***P-*value**	**Odds ratio**	***p-*value**
**Home environment**						
Parent warmth	0.569	0.003	0.528	0.010	0.762	0.072
Parent acceptance	0.446	0.000	0.570	0.018	0.762	0.036
Parent consistency	0.142	0.000	0.498	0.001	0.460	0.000
**School environment**						
Peer relationship	0.059	0.000	0.118	0.000	0.357	0.000
Teacher relationship	0.141	0.000	0.357	0.000	0.326	0.000

*The groups written next to ‘VS.' are used as reference groups in each regression*.

## Discussion

In the current study, we performed a latent profile analysis on risk profiles of Korean adolescents with the relation of contextual factors. Before describing some meaningful implications for future research and practice, we emphasize that the analysis was only derived from the 1st year data of KCYPS 2018, and the results should be taken into consideration with caution.

### Risk Profiles of Korean Adolescents

We could identify four risk profiles of Korean adolescents: the high-risk group, the M-ACA risk group, the M-SEB risk group, and the low-risk group. These profiles were determined based on the major risk factors faced by Korean adolescents, encompassing social, emotional, behavioral, and academic domains. This analytic result supports the previous study, having classified the latent classes of students at-risk rated by their teachers; the overall high-risk group, social and behavioral risk group, and the academic risk group (33). The percentage of students included in the high-risk group was 16.8%, which corroborates the typical proportion of students (15–25%) who should be referred to the selective interventions in the MTSS as well (4, 45). Therefore, it can be concluded that through LPA, we were able to identify students in the high-risk group as youths at-risk who should be referred to more intensive instructions within MTSS. Youth at-risk identified from the current study showed the highest levels of risks among four risk profiles across social, emotional, behavioral, and academic domains. Through this result, MTSS, a comprehensive framework aimed to address the interplay of social, emotional, behavioral, and academic functioning in the classroom (24, 25), can be suggested as the most suitable educational service for youth at-risk.

It was also found that the M-SEB group showed higher risks in aggression, social withdrawal, and depression than in academic behavior and academic motivation, while the M-ACA group indicated the opposite result. Through this result, risk factors incorporated in the latent profile analysis can be easily classified into two clusters–one for the SEB risks and the other for the academic risks, and we can conclude that some students need more SEB support than academic one whereas, others need more academic support than SEB one. Therefore, it can be suggested that instructions in MTSS should be planned in two tracks, with one with services for academic functioning and the other for SEB adaptation. According to Briesch et al. (46), conceptual models for MTSS are often configured as a “double triangle,” encompassing tiered interventions to enhance both academic and behavioral competencies. During the actual implementation, however, the nature of guidelines for MTSS mainly was based on academic domains of services, which led educators to assume that procedures for addressing academic challenges are identically applicable to SEB domains as well (47). We should note that there are some critical differences between the actual implementation of MTSS in academic and SEB domains in terms of types of interventions, tools and frequency for assessments, and criteria for assessing response to interventions (46, 48). Therefore, it is highly required to build systematic assessment and intervention systems centering on the unique characteristics of SEB challenges which can be distinguished from academic ones.

Luckily, recent studies attempted to develop standardized assessments and intervention programs customized to address student's SEB challenges within MTSS. For instance, Harrell-Williams et al. (49) developed a Behavioral and Emotional Screening System (BESS) to screen students who have problems in behavior and emotions and refer them to tier 2 interventions. Kilgus et al. (9) also devised a universal screening assessment tool named Social, Academic, and Emotional Behavior Risk Screener (SAEBRS), whose result shows student's social and emotional behavior level extracted from academic behavior. Additionally, there have been developed some tier 2 interventions targeting students with SEB challenges, such as Check-In/Check-Out (50) for improving social and behavioral competencies and the Resilience Education Program (51) for enhancing emotional competencies and addressing internalizing problems.

Unlike other risk factors, however, the level of attention deficit in the M-SEB group had no significant difference from that in the M-ACA group. This result demonstrates that the attention problems of students are highly likely to have comorbidity both with SEB and academic challenges, being the typical characteristics of defining youth at-risk. Therefore, adolescents suffering from being immersed in school lessons need to be referred to detailed assessments as soon as possible in order for the early identification of youth at risk.

### Contextual Factors Affecting Risk Profiles

Home and school environmental factors, which were included as covariates in the LPA, significantly influenced the risk profiles of Korean adolescents. Specifically, the present study suggested that compared to both low and M-ACA risk groups, the students in the high-risk group were impacted by all contextual factors included in the research model. It was also found that compared to the M-SEB risk group, parental acceptance, parental consistency, peer and teacher relationships were also significantly affected the probability of deciding youth at-risk. These results support numerous former studies investigating the effect of home and environmental variables such as relationships with parents, peers, and teachers on school adjustment as well as the overall well-being of students (21, 22, 52, 53). According to Kim et al. (54), the inconsistent parenting attitude mediated the effect of school adjustment on student's life satisfaction, which consequently led students to low satisfaction in their lives overall. Furthermore, it was also demonstrated that school environmental factors such as relationships with peers and teachers had significant influences on students' mental health as well as overall self-concept that are crucial to school and adulthood adjustment (21, 23). Based on the results of former and current studies, we can thus conclude that home and school environmental variables, including relationships with parents, peers, and teachers, are highly recommended to be considered when planning the educational support for youth at-risk.

Although it is imperative to consider the necessity of adaptive instructions with a responsive decision-making process for each student within MTSS (27, 55), the adaptation has been implemented in a limited way. Majeika et al. (55) described two ways of adaptation: a horizontal adaptation which is based on student characteristics and contextual factors, and a vertical adaptation grounded on data indicating a student's response to intervention. As MTSS is initially designed to develop tiered intervention programs based on student's response to intervention, it has been common to consider a vertical adaptation process. However, teachers have often ignored the effect of contextual factors on student's performances and interventions within MTSS (27). In addition to the result of the current study that contextual factors have significant effects on deciding youth at-risk, we now have to take a more active stance toward horizontal adaptations when implementing MTSS in school settings.

One of the most effective ways to initiate horizontal adaptations is to manage social dynamics surrounding each student and classroom (29). Social dynamics indicate the social roles and relationships with significant others, and unhealthy social dynamics may inhibit students' performance despite being able to perform it (56). Hence, teachers must be accurately aware of social dynamics in classrooms and manage them to help operate instructional practices (57). The social dynamics management is thus aimed to provide students with opportunities to develop relationships with peers who support and complement one another's strengths and the development of new skills, beliefs, and values by teachers being attuned to the peer culture and social hierarchy and monitoring the dynamics of power in classrooms (32). In order to accomplish these goals, Farmer et al. (29) suggested a few strategies to manage classroom social dynamics as follows successfully: using information about the peer system to help guide classroom arrangement and behavior management strategies; monitoring whether students feel safe and socially comfortable in the classroom; changing contexts to prevent negative roles, interactive patterns, and social relationships. Additionally, as interactions with parents became significant factors predicting youth at-risk, it is also necessary to intervene in social dynamics at home as well as in the classrooms. Kim (58) articulated that counseling and intervention strategies for parents of students at-risk should be different across students' major problems (e.g., low academic motivation and competencies, depressive symptoms, social withdrawal, and other hidden handicaps) and parenting types (e.g., controlling vs. permissive, autocratic vs. pushover). Although there have been several guidelines for managing social dynamics in classrooms and at home, we still need to develop how these strategies can be flexibly incorporated in general and targeted educational services within MTSS (32). This is what educators should strive for.

### Limitations and Suggestions for Future Research

There were some limitations related to the data sources we used in this research. Due to the limited arrangement of variables included in the data, the number of risk factors was only six, which may be marginal to encompass all types of difficulties faced by adolescents. For example, it is more plausible to incorporate each student's actual academic performance to assess academic challenges accurately. However, academic motivation and behavior scales were alternatively used to identify students' academic risks because KCYPS 2018 did not provide information on students' actual academic performance. Similarly, although temperament risk factors such as effortful control may also significantly impact deciding youth at-risk (19, 20), they were not able to be included in the present study. The contextual factors included in this study were also limited. Other than teacher, peer, and parent relationships, the social-economic status of each family may have significantly influenced the development of risk factors. The following study thus needs to design more extensive models to identify youth at-risk by adding other risk and contextual variables.

Upon this, we only used the first-year data of KCYPS since participants were not obligated to report their levels of risks during the survey of the following year. In other words, there were a significant number of missing data in the second-year data, which led us to decide to use only the first-year data from middle school 1st graders. The risk profiles of middle school 1st graders may not reflect the general tendency of adolescents of all ages (12–18). Therefore, future research should incorporate older youths in the analysis to confirm the findings from the current research.

Lastly, the current study's findings can be more robust if corroborated by biological evidence such as changes in area and degree of brain activation observed by EEG or fMRI. Due to a lack of prior research discovering the effect of social relationships with peers, teachers, or parents on the biological markers of youth at-risk, the present study had limitations in predicting possible outcomes of horizontal adaptations. Hence, future studies need to demonstrate objective biomarkers that can be influenced by social dynamics of youth at-risk and predict expecting outcomes of horizontal adaptation in the long term.

## Data Availability Statement

The data analyzed in this study is subject to the following licenses/restrictions: The dataset is from the national survey by the National Youth Policy Institute in the Republic of Korea in 2018, and the authors do not have the right to release the dataset. Requests to access these datasets should be directed to JH, lifewizard@snu.ac.kr.

## Ethics Statement

Ethical review and approval was not required for the study on human participants in accordance with the local legislation and institutional requirements. Written informed consent to participate in this study was provided by the participants' legal guardian/next of kin.

## Author Contributions

DK writing of the first and final draft of the manuscript, interpretation of analysis, concept and design of the research, and final approval for publication. JL writing of the first and final draft of the manuscript, acquisition of data, interpretation of analysis, concept and design of the research, and final approval for publication. All authors contributed to the article and approved the submitted version.

## Funding

This work was supported by the Ministry of Education of the Republic of Korea and the National Research Foundation of Korea (NRF-2020S1A3A2A02103411).

## Conflict of Interest

The authors declare that the research was conducted in the absence of any commercial or financial relationships that could be construed as a potential conflict of interest.

## Publisher's Note

All claims expressed in this article are solely those of the authors and do not necessarily represent those of their affiliated organizations, or those of the publisher, the editors and the reviewers. Any product that may be evaluated in this article, or claim that may be made by its manufacturer, is not guaranteed or endorsed by the publisher.

## References

[1] Erikson EH. The problem of ego identity. J Am Assoc. (1956) 4:56–121. 10.1177/000306515600400104 13286157

[2] Kwon HY. Qualitative analysis on psychosocial factors of an actual counseling cases from youth companion counselor to the youth in crisis. J Humanit. (2013) 33:151–81.

[3] Resnick G Burt MR. Youth at risk: definitions and implications for service delivery. Am J Orthopsychiatry. (1996) 66:172–88. 10.1037/h0080169 8860747

[4] Evans P. Community-based approaches and cross-sectoral partnerships for youths at risk in OECD countries. National Youth Commissions (Eds.), Building a community-based safety net for youths at risk: International trends of youth policies and the policy tasks in Korea. Seoul (2005). p. 10–6.

[5] Dryfoos JG. Adolescents at Risk. New York, NY: Oxford University Press (1990).

[6] Massey OT Armsrong K Boroughs M Henson K McCash L. Mental health services in schools: a qualitative analysis. Psychol Sch. (2005) 42:361–72. 10.1002/pits.20063

[7] Scheel MJ Madabhushi S Backhaus A. The academic motivation of at-risk students in a counseling prevention program. Couns Psychol. (2009) 37:1147–78. 10.1177/0011000009338495

[8] Han SM. The relationships between the academic motivation variables, cognitive strategies and academic achievement. Korean J Educ Psychol. (2004) 18:329–50.

[9] Kilgus SP Eklund K von der Embse NP Taylor CN Sims WA. Psychometric defensibility of the social, academic, and emotional behavior risk screener (SAEBRS) teacher rating scale and multiple gating procedure within elementary and middle school samples. J School Psychol. (2016) 58:21–39. 10.1016/j.jsp.2016.07.001 27586068

[10] Shaw M Hodgkins P Caci H Young S Kahle J Woods AG. A systematic review and analysis of long-term outcomes in attention deficit hyperactivity disorder: effects of treatment and non-treatment. BMC Med. (2012) 10:99. 10.1186/1741-7015-10-99 22947230 PMC3520745

[11] Hechtman L. Predictors of long-term outcome in children with attention-deficit/hyperactivity disorder. Pediatr Clin North Am. (1999) 46:1039–52. 10.1016/S0031-3955(05)70171-1 10570704

[12] Sharma MK Marimuthu P. Prevalence and psychosocial factors of aggression among youth. Indian J Psychol Med. (2014) 36:48–53. 10.4103/0253-7176.127249 24701010 PMC3959019

[13] Oh W Rubin KH Bowker JC Booth-LaForce C Rose-Krasnor L Laursen B. Trajectories of social withdrawal from middle childhood to early adolescence. J Abnorm Child Psychol. (2008) 36:553–66. 10.1007/s10802-007-9199-z 18193479 PMC3674845

[14] Rubin KH. The waterloo longitudinal project: correlates and consequences of social withdrawal from childhood to adolescence. In: Rubin KH Asendorpf JB, Editors. Social Withdrawal, Inhibition, & Shyness in Childhood. Hillsdale, NJ: Erlbaum (1993). p. 291–314.

[15] Bowker A Bukowski W Zargarpour S Hoza B. A structural and functional analysis of a two-dimensional model of withdrawal. Merrill Palmer Q. (1998) 44:447–63.

[16] Rubin KH Chen X Hymel S. The socio-emotional characteristics of extremely aggressive and extremely withdrawn children. Merrill Palmer Q. (1993) 39:518–34.

[17] Birmaher B Ryan ND Williamson DE Brent DA Kaufman J Dahl RE. Childhood and adolescent depression: a review of the past 10 years. part I. J Am Acad Child Adolesc Psychiatry. (1996) 35:1427–39. 10.1097/00004583-199611000-00011 8936909

[18] Field T Miguel D Sanders C. Adolescent depression and risk factors. Adolescence. (2001) 36:491–8. 11817630

[19] Santens E Claes L Dierckx E Dom G. Effortful control: a transdiagnostic dimension underlying internalizing and externalizing psychopathology. Neuropsychobiology. (2020) 79:255–69. 10.1159/000506134 32106115

[20] Veronneau M-H Racer KH Fosco GM Dishion TJ. The contribution of adolescent effortful control to early adult educational attainment. J Educ Psychol. (2014) 106:730–43. 10.1037/a0035831 25308996 PMC4191676

[21] Lim J. Mental health of middle school low achieving students and the impact of personal and environmental variables. J Learn Center Curric Instruct. (2020) 20:377–403. 10.22251/jlcci.2020.20.23.377

[22] Kim D Lim J. The mediating effect of mental health problems on correlation between self-concept and school adjustment of adolescents: Multi-group analysis between low achievers and students without disabilities. Korean J Learn Disabil. (2020) 17:107–28. 10.47635/KJLD.2020.17.3.107

[23] Kim D Lim J. The effect of home and school environment on self-concept profile of middle school low achievers. SNU J Educ Res. (2021) 30:37–58. 10.54346/sjer.2021.30.1.37

[24] Lane KL Carter EW Jenkins A Dwiggins L Germer K. Supporting comprehensive, integrated, three-tiered models of prevention in schools: administrators perspectives. J Posit Behav Interv. (2015) 17:209–22. 10.1177/1098300715578916

[25] Kim EK Anthony CJ Chafouleas SM. Social, emotional, and behavioral assessment within tiered decision-making frameworks: advancing research through reflections on the past decade. School Psych Rev. (2021) 51:1–5. 10.1080/2372966X.2021.1907221

[26] Chen C Farmer TW Hamm JV Brooks DS Lee D Norwalk K. Emotional and behavioral risk configurations, students with disabilities, and perceptions of the middle school ecology. J Emot Behav Disord. (2020) 28:180–92. 10.1177/1063426619866829

[27] Farmer TW Sutherland KS Talbott E Brooks D Norwalk K Huneke M. Special educators as intervention specialists: dynamic systems and the complexity of intensifying intervention for students with emotional and behavioral disorders. J Emot Behav Disord. (2016) 24:173–86. 10.1177/1063426616650166

[28] Sailor W. Advances in school-wide inclusive school reform. Remedial Spec Educ. (2015) 36:94–9. 10.1177/0741932514555021

[29] Farmer TW Hamm JV Dawes M Barko-Alva K Cross JR. Promoting inclusive communities in diverse classrooms: teacher attunement and social dynamics management. Educ Psychol. (2019) 54:286–305. 10.1080/00461520.2019.1635020

[30] Sutherland KS Farmer TW Kunemund RL Sterrett BI. Learning, behavioral, and social difficulties within MTSS: a dynamic perspective of intervention intensification. In Young ND Bonanno-Sotiropoulos K Citro TA, Editors. Paving the Pathway For Educational Success: Effective Classroom Interventions For Students With Learning Disabilities. New York, NY: Rowman & Littlefield (2018). p. 15–33.

[31] Cairns RB Cairns BD. Lifelines and Risks: Pathways of Youth in Our Time. New York, NY: Harvester Wheatsheaf (1994).

[32] Farmer TW Bierman KL Hall CM Brooks DS Lee DL. Tiered systems of adaptive supports and the individualization of intervention: Merging developmental cascades and correlated constraints perspectives. J Emot Behav Disord. (2021) 29:3–13. 10.1177/1063426620957651

[33] Cho A Yoo I Lee Y Hwang J Choi S. Classifying latent classes and analyzing the characteristics of at-risk learners in blind spot of education across school levels. Korea J Learn Disabil. (2021) 18:155–78. 10.47635/KJLD.2021.18.1.155

[34] Bak B-G Roh S-U Kim J-A Hwang J-S. Development and validation of the academic helplessness scale. J Child Educ. (2015) 24:5–29. 10.17643/KJCE.2015.24.4.01

[35] Cho B-H Lim K-H. Development and validation of emotional or behavioral problems scale. Korean J Counsel Psychotherapy. (2003) 15:729–46.

[36] Kim S-H Kim K-Y. Development of behavior problem scale for children and adolescence. J Korean Home Manag Assoc. (1998) 16:155–66. 10.5124/jkma.1998.41.2.155

[37] Kim G-I Kim J-H Won H-T. Korean Symptom Check List. Seoul: Jungangjeoksung-chulpansa (1984).

[38] Kim T Lee E. Validation of the Korean version of parents as social context questionnaire for adolescents: PSCQ_KA. Korean J Youth Stud. (2017) 24:313–33. 10.21509/KJYS.2017.03.24.3.313

[39] Bae SM Hong JY Hyun MH. A validation study of the peer relationship quality scale for adolescents. Korean J Youth Stud. (2015) 22:325–44.

[40] Kim JB Kim NH. Validation of student-teacher attachment relationship scale (STARS) as a basis for evaluating teachers' educational competencies. Korean J Educ Psychol. (2009) 23:697–714.

[41] Lim DH Ryu H Jin B. A latent class analysis of older workers' skill proficiency and skill utilization in South Korea. Asia Pac Educ Rev. (2020) 21:365–78. 10.1007/s12564-020-09632-2

[42] Muthen B Muthen LK. Integrating person-centered and variable-centered analyses: growth mixture modeling with latent trajectory classes. Alcohol Clin Exp Res. (2000) 24:882–91. 10.1111/j.1530-0277.2000.tb02070.x 10888079

[43] Asparouhov T Muthen B. Auxiliary variables in mixture modeling: three-step approaches using Mplus. Struct Equ Model. (2014) 21:329–41. 10.1080/10705511.2014.915181

[44] Lubke G Muthen B. Performance of factor mixture models as a function of model size, covariate effects, and class-specific parameters. Struct Equ Model. (2007) 14:26–47. 10.1080/10705510709336735

[45] Kim D. Basic Academic Skills Assessment: Reading. Seoul: Hakjisa (2000).

[46] Briesch AM Chafouleas SM Nissen K Long S. A review of state-level procedural guidance for implementing multi-tiered systems of support for behavior (MTSS-B). J Posit Behav Interv. (2020) 22:131–44. 10.1177/1098300719884707

[47] Bruhn AL Wehby JH Hasselbring TS. Data-based decision making for social behavior: setting a research agenda. J Posit Behav Interv. (2020) 22:116–26. 10.1177/1098300719876098

[48] Hawken LS Vincent CG Schumann J. Response to intervention for social behavior: challenges and opportunities. J Emot Behav Disord. (2008) 16:213–25. 10.1177/1063426608316018

[49] Harrell-Williams LM Raines TC Kamphaus RW Dever BV. Psychometric analysis of the BASC-2 behavioral and emotional screening system (BESS) student form: results from high school student samples. Psychol Assess. (2015) 27:738–43. 10.1037/pas0000079 25642926

[50] Kilgus SP Fallon LM Feinberg AB. Function-based modification of check-in/check-out to influence escape-maintained behavior. J Applied School Psychol. (2016) 32:24–45. 10.1080/15377903.2015.1084965

[51] Kilpatrick KD Kilgus SP Eklund K Herman KC. An evaluation of the potential efficacy and feasibility of the resilience education program: a tier 2 internalizing intervention. School Mental Health. (2021) 13:376–91. 10.1007/s12310-021-09428-8

[52] Bireda AD Pillay J. Perceived parent-child communication and well-being among Ethiopian adolescents. J Adolesc Youth. (2018) 23:109–17. 10.1080/02673843.2017.1299016

[53] Dubois DL Eitel SK Felner RD. Effects of family environment and parent-child relationships on school adjustment during the transition to early adolescence. J Marriage Fam. (1994) 56:405–14. 10.2307/353108

[54] Kim SK Park MK An JS. The influence of school adjustment on life satisfaction of middle school students: the moderating effects of perceived parental behavior. J Youth Welfare. (2014) 16:163–82.

[55] Majeika CE Bruhn AL Sterrett BI McDaniel S. Reengineering tier 2 interventions for responsive decision making: an adaptive intervention process. J Appl School Psychol. (2020) 36:111–32. 10.1080/15377903.2020.1714855

[56] Coussi-Korbel S Fragaszy DM. On the relation between social dynamics and social learning. Anim Behav. (1995) 50:1441–53. 10.1016/0003-3472(95)80001-823596900

[57] Farmer TW Reinke W Brooks DS. Managing classrooms and challenging behavior: theoretical considerations and critical issues. J Emot Behav Disord. (2014) 22:67–73. 10.1177/1063426614522693

[58] Kim D. Counseling Parents of At-Risk Learners. Seoul: Park Young Story (2020).

