# Corrigendum: Risk Profiles of Korean Adolescents in Relations With Contextual Factors: Implications for Multi-Tiered Systems of Support

**DOI:** 10.3389/fpsyt.2022.902002

**Published:** 2022-05-12

**Authors:** Dongil Kim, Jin Hyung Lim

**Affiliations:** Department of Education, Seoul National University, Seoul, South Korea

**Keywords:** youth at-risk, risk profiles, contextual factors, MTSS, KCYPS 2018

In the original article, there was an error in the supplementary material. Although the authors declared that they do not have the right to release the dataset in the Data Availability Statement, the raw dataset was mistakenly incorporated in the supplementary material. New supplementary material has been published which does not contain the data that the authors do not have the right to release.

The authors apologize for this error and state that this does not change the scientific conclusions of the article in any way. The original article has been updated.

## Publisher's Note

All claims expressed in this article are solely those of the authors and do not necessarily represent those of their affiliated organizations, or those of the publisher, the editors and the reviewers. Any product that may be evaluated in this article, or claim that may be made by its manufacturer, is not guaranteed or endorsed by the publisher.

